# Malaria incidence and mortality in Zimbabwe during the COVID-19 pandemic: analysis of routine surveillance data

**DOI:** 10.1186/s12936-021-03770-7

**Published:** 2021-05-24

**Authors:** Samuel Gavi, Oscar Tapera, Joseph Mberikunashe, Mufaro Kanyangarara

**Affiliations:** 1 Department of Epidemiology and Biostatistics, Arnold School of Public Health, University of South Carolina, 915 Greene Street, Columbia, SC 29208 USA; 2 Sadtap Health Research Institute, Harare, Zimbabwe; 3 National Malaria Control Programme, Ministry of Health and Child Care, Harare, Zimbabwe

**Keywords:** Malaria, COVID-19, Zimbabwe, Sub-Saharan Africa

## Abstract

**Background:**

The coronavirus disease 2019 (COVID-19) pandemic has posed a unique challenge to health care systems globally. To curb COVID-19 transmission, mitigation measures such as travel restrictions, border closures, curfews, lockdowns, and social distancing have been implemented. However, these measures may directly and indirectly affect the delivery and utilization of essential health services, including malaria services. The suspension of indoor residual spraying (IRS) and insecticide-treated net (ITN) distribution, shortages of malaria commodities, and reduced demand for health services have hindered the continued delivery of malaria services. The overall goal of this analysis was to describe the trends in malaria incidence and mortality in Zimbabwe prior to and during the pandemic to understand the consequences of COVID-19-related changes in the delivery and utilization of malaria services.

**Methods:**

Monthly data on the number of malaria cases and deaths by district for the period January 2017 to June 2020 were obtained from the national health management information system (HMIS). District-level population data were obtained from the 2012 Census. Malaria incidence per 1000 population and malaria deaths per 100,000 population were calculated for 2017, 2018, 2019, and 2020 and mapped to describe the spatial and temporal variation of malaria at the district level.

**Results:**

Compared to the same period in 2017, 2018 and 2019, there was an excess of over 30,000 malaria cases from January to June 2020. The number of malaria deaths recorded in January to June 2020 exceeded the annual totals for 2018 and 2019. District level maps indicated that areas outside high malaria burden provinces experienced higher than expected malaria incidence and mortality, suggesting potential outbreaks.

**Conclusions:**

The observed surge in malaria cases and deaths in January to June 2020 coincided with the onset of COVID-19 in Zimbabwe. While further research is needed to explore possible explanations for the observed trends, prioritizing the continuity of essential malaria services amid the COVID-19 pandemic remains crucial.

**Supplementary Information:**

The online version contains supplementary material available at 10.1186/s12936-021-03770-7.

## Background

The coronavirus disease 2019 (COVID-19) pandemic has posed a unique challenge to health care systems globally. To curb COVID-19 transmission, mitigation measures such as travel restrictions, border closures, curfews, lockdowns, and social distancing have been implemented. However, these measures may directly and indirectly affect the delivery and utilization of essential health services, including malaria services. The suspension of indoor residual spraying (IRS) and insecticide-treated net (ITN) distribution, shortages of malaria commodities, and reduced demand for health services have hindered the continued delivery of malaria services. The overall goal of this analysis was to describe the trends in malaria incidence and mortality in Zimbabwe prior to and during the pandemic to understand the consequences of COVID-19-related changes in the delivery and utilization of malaria services.

The first official cases of COVID-19 were reported in Wuhan, China in December 2019 after an outbreak of pneumonia of unknown origin was identified [1]. On 30 January, 2020, the outbreak was declared a public health emergency of international concern (PHEIC) by the World Health Organization (WHO) [2]. Two weeks later, the first case of COVID-19 was reported on the African continent in Egypt [3]. In response to the looming public health crisis, African governments introduced regulations and restrictions such as travel bans, border closures, curfews, lockdowns, and social distancing [4]. While these mitigation measures were aimed at slowing down the spread of COVID-19 and preventing healthcare systems from becoming overwhelmed, there have been wide-reaching unintended consequences on health systems. The demand for essential health services has been adversely affected by stay-at-home orders, stigma and fear of contracting COVID-19 infection, travel restrictions, increased financial barriers and misinformation about COVID-19 [5–7]. The provision of routine health services has been disrupted by the diversion of limited resources and health care workers for COVID-19 prevention and control efforts, the occupational risk faced by health care workers, the lack of personal protective equipment, and breakdowns in procurement and supply chains for medicines and commodities [7, 8]. Similar reductions in the demand and provision of essential health services were observed during the 2014–2016 Ebola outbreak in West Africa [9].

For national malaria control programmes, the reduced access to and availability of malaria services, disruptions in the production and supply of malaria commodities, and the suspension of IRS and ITN distribution campaigns have hindered the continued provision of malaria services during the COVID-19 pandemic [10]. Several modelling analyses have assessed the potential impact of disruptions in health service provision on malaria morbidity and mortality. Findings from these analyses have asserted that lack of continuity and disruption of malaria programmes could cause a COVID-19-induced malaria crisis, potentially reversing the gains towards malaria control and elimination [11–14]. More devastating effects of the pandemic are expected in Africa because of the disproportionate burden of infectious diseases, including malaria, weaker health systems and limited financial and human resources for health [11, 12]. One modelling analysis projected that if the distribution of ITNs was suspended and access to anti-malarial medicines reduced by 75%, an additional 769,000 malaria deaths would occur in sub-Saharan Africa in 2020 [14]. Another modelling analysis estimated that in malaria-endemic areas, the disruption of planned ITN distribution campaigns, because of the COVID-19 pandemic, could cause a 36% increase in malaria deaths in the next five years [12]. COVID-related reductions or suspensions of ITN and IRS campaigns, which are the cornerstone of malaria vector control in Africa, were projected to give rise to the largest number of additional malaria deaths [11–14]. These estimates most likely underestimate the potential excess mortality as the underlying models do not account for disruptions in the delivery of other life-saving malaria interventions, such as seasonal malaria chemoprevention (SMC) and intermittent preventative treatment in pregnancy (IPTp) [15]. However, these initial estimates inform pandemic response by forecasting morbidity and mortality, estimating healthcare system requirements and assessing the effectiveness of various containment and mitigation strategies.

Malaria transmission within Zimbabwe is spatially heterogenous; areas from the northwest to southeast borders of the country are characterized as high malaria-risk zones, while, areas along the central plateau and in the southwest of the country experience little to no malaria transmission [16]. The rainy season typically runs from October to April, with increasing average annual rainfall from the west to the east of the country [17]. Half (50%) of the population is at risk of malaria infection [18]. For vector control, the National Malaria Control Programme (NMCP) implements a two-pronged approach that targets the deployment of either ITNs or IRS to malarious districts. In moderate and high transmission areas, 51% of households own at least one ITN for every two household members, and 85% of households own at least one ITN and/or are protected by IRS, showing high levels of protection [19].

**Fig. 1:**
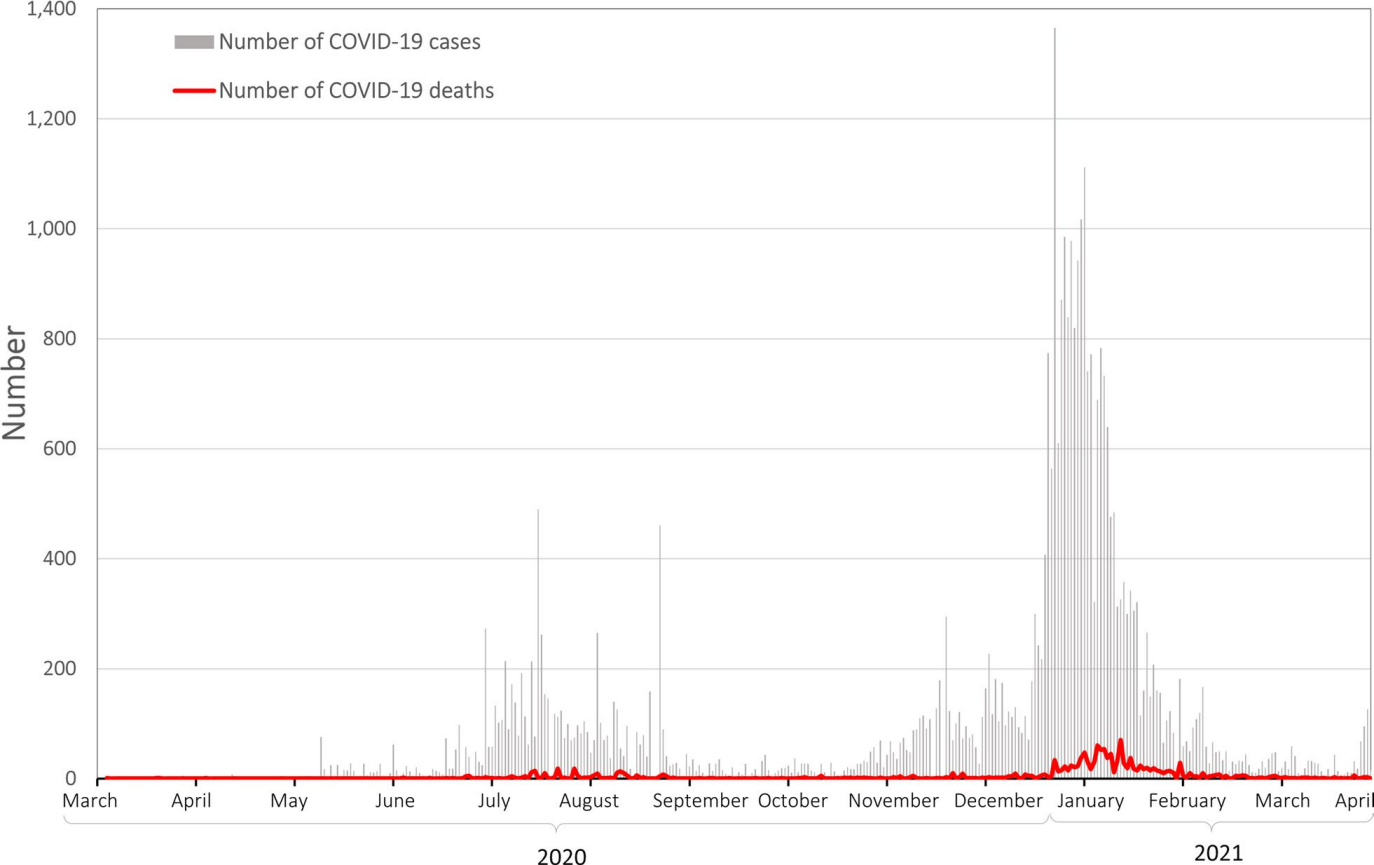
Confirmed COVID-19 cases and deaths in Zimbabwe, March 2020–April 2021

In Zimbabwe, the first official case of COVID-19 was reported on 20 March, 2020 [20] (Fig. 1). The delay in identifying the first COVID-19 cases, despite several people exhibiting symptoms after international travel has been attributed to the lack of COVID-19 testing capacity and resources [21, 22]. In response to COVID-19, the Government of Zimbabwe introduced public health measures to curb the spread of disease on 30 March, 2020 [6, 21, 22]. These measures included closure of borders, restrictions on in-country travel, bans on public gatherings, the closure of schools, colleges and universities, and the designation of quarantine and isolation facilities for suspected and confirmed COVID-19 cases [22, 23]. There was a peak in the number of COVID-19 cases and deaths in September and October 2020 (Fig. 1). Since December 2020, the country has been experiencing a second wave of COVID-19 with a higher transmission rate [24]. The second wave coincides with the onset of the rainy season and the start of increased malaria transmission season. As of 21 April, 2021, a cumulative 37,859 confirmed COVID-19 cases and 1,553 deaths had been reported in Zimbabwe according to the WHO (Fig. 1) [24].

Understanding the indirect consequences of the COVID-19 pandemic remains key to developing health system resilience, allocating limited resources to pandemic response while maintaining essential health services, and sustaining efforts to control malaria and address other health priorities. Therefore, monitoring malaria morbidity and mortality is essential to tracking the consequences of COVID-19-related changes to the delivery and utilization of malaria services [25]. The overall goal of this analysis was to describe the trends of malaria incidence and mortality in Zimbabwe prior to and during the COVID-19 pandemic to inform program planning and decision-making. Using routine data from the national health management information system (HMIS), this analysis described the spatial and temporal trends of malaria incidence and mortality from January 2017 to June 2020. These findings may help improve understanding of the indirect health consequences of the pandemic and draw attention to the need for sustained efforts to control malaria amidst the pandemic.

## Methods

Zimbabwe is a land-locked country that shares borders with 4 other malaria-endemic countries: Zambia in the north, Mozambique in the east, South Africa in the south; Botswana in the west and southwest. The country has a population of 16.2 million people, and two-thirds of the population live in rural areas [26]. The country is divided into 10 provinces, comprising 62 districts. The delivery of essential health services, including malaria diagnosis and treatment is decentralized. According to the 2015 Zimbabwe Demographic and Health Survey (DHS), 77% of live births were delivered at a health facility and 51% of children under five years with a fever were taken to a health facility for treatment [27].

### Data and analysis

This was a retrospective descriptive study of malaria incidence and mortality in Zimbabwe from January 2017 to June 2020. Passive malaria surveillance data are routinely collected from all public and private health facilities and submitted to the HMIS regularly. The malaria data are relatively complete [28]. For this analysis, confirmed malaria cases and deaths data aggregated by month and district were used. Given weekly fluctuations in malaria cases and deaths, monthly data was used for this analysis. This analysis used data up to 30 June, 2020 to ensure the inclusion of the peak malaria transmission season for 2020.

Malaria cases and deaths were aggregated by province and year (2017, 2018, 2019, 2020), and stratified by age (< 5 years and ≥ 5 years) and gender (male and female). Using district population counts from the 2012 Census adjusted for annual population growth rate (1.1%) [26], malaria incidence per 1000 population and malaria mortality per 100,000 population were calculated. Changes in malaria cases and deaths during the pandemic were evaluated by comparing the observed values for January-June 2020 to expected values based on previous years. The expected values were calculated by taking the mean of values for the same months in 2017, 2018 and 2019. Maps of malaria incidence and mortality by year were used to describe the spatial variation at the district level. Data management and analysis were conducted using STATA 15 (Stata Corporation, College Station, TX, USA) and maps were generated using ArcMap 10.5.1 (ESRI, Redlands, CA, USA).

### Ethical considerations

Aggregate-level data on malaria cases and deaths from districts were used. Ethical clearance was not required as no individual personal identifying information was used.

## Results

**Fig. 2:**
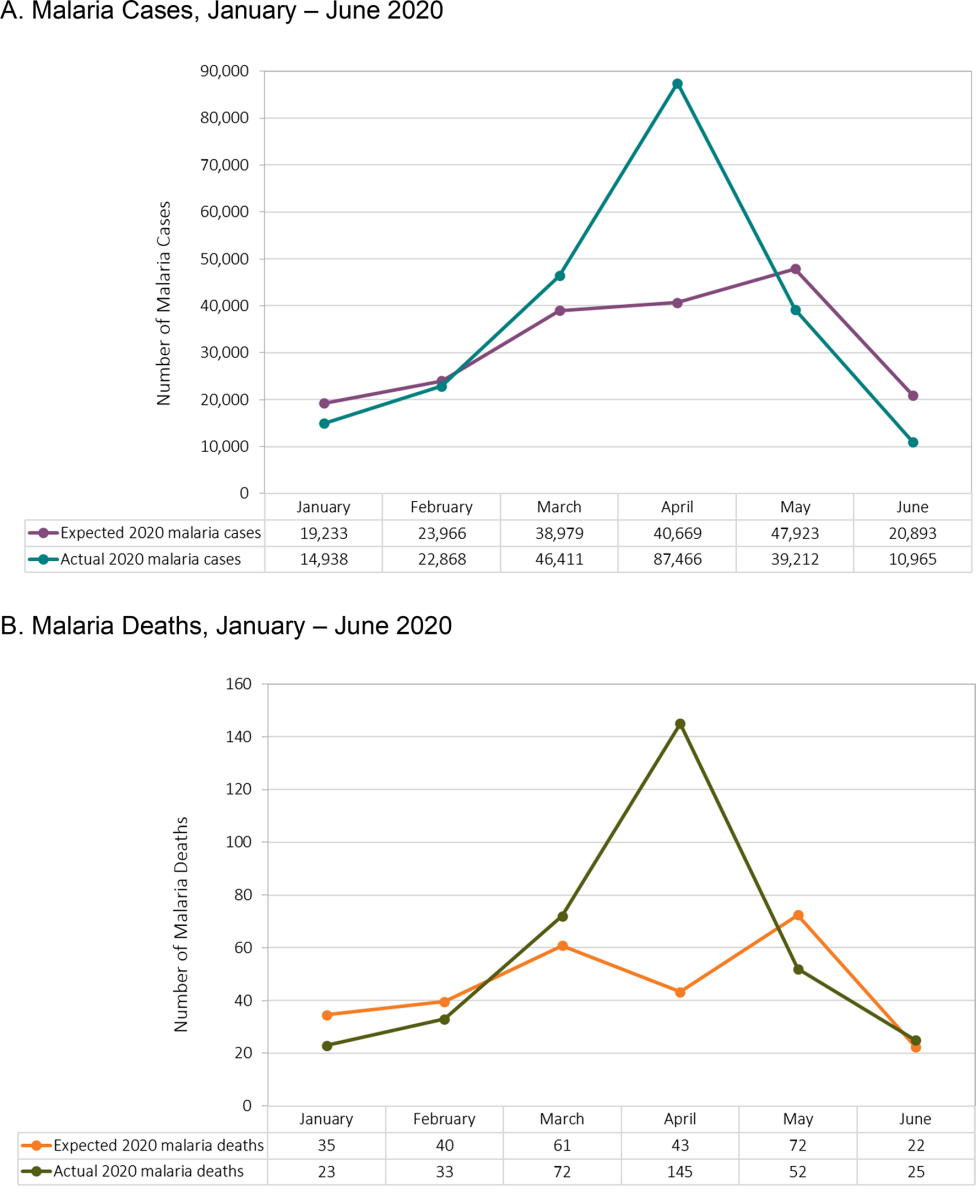
Changes in malaria cases and deaths in Zimbabwe, January–June 2020

At national level, the annual number of confirmed malaria cases decreased by 41% from 316,431 in 2017 to 186,556 in 2019 (Table 1). The lowest number of malaria cases was observed in 2018. Of the reported cases, about 50% were among females (Additional file 1: Figure S1). The proportion of malaria cases among children under five years of age fluctuated between 10 and 20% (Additional file 2: Figure S2). Across all years, Manicaland, Mashonaland Central, Mashonaland East and Masvingo provinces had the highest number of confirmed malaria cases and accounted for over 85% of the national burden of malaria cases (Table 1). As of 30 June, 2020, there were 605 cumulative COVID-19 cases and 17 COVID-19 deaths. Harare, the capital city had the highest number of cases. Between January and June 2020, which coincided with the peak malaria transmission season, there was a surge in malaria cases as 221,860 confirmed malaria cases were reported. In January and February 2020, the number of malaria cases reported was fairly similar to the expected number of malaria cases based on previous years (Fig. 2A). However, there was a higher than expected increase in malaria cases in March 2020, which coincided with detection of the first COVID-19 case in Zimbabwe. In April 2020, malaria cases were more than double the expected number. Overall, between January and June 2020, the number of malaria cases exceeded the expected number by 30,197 cases (16%).

**Table 1:** Number of confirmed malaria and COVID-19 cases and deaths in Zimbabwe, January 2017–June 2020

	Malaria cases				Malaria deaths				Cumulative COVID-19 cases	Cumulative COVID-19 deaths
	2017	2018	2019	January–June 2020	2017	2018	2019	January–June 2020	March–June 2020	March–June 2020
National	316,431	184,427	186,556	221,860	531	232	311	350	605	7
Province										
Bulawayo	161	104	73	59	45	5	13	8	64	3
Harare	3167	1614	1977	891	57	34	59	20	225	2
Manicaland	98,093	48,783	57,909	67,841	140	52	73	97	29	0
Mashonaland Central	70,233	37,400	51,776	50,996	47	24	42	54	10	0
Mashonaland East	69,146	35,962	39,208	47,293	63	32	39	62	55	0
Mashonaland West	11,720	13,158	12,957	21,071	20	18	23	36	41	1
Masvingo	54,224	39,314	17,673	24,978	118	47	44	42	53	0
Matabeleland North	2303	3357	1916	2048	9	5	5	4	17	0
Matabeleland South	5480	2529	997	2896	21	10	9	15	54	0
Midlands	1904	2206	2070	3787	11	5	4	12	57	1

Like the pattern observed for malaria cases, the number of malaria deaths dropped by 41% between 2017 and 2019 (Table 1) Additional file 3: Figure S3. However, the declining trend was reversed in 2020 when the number of deaths reported between January and June (n = 350) exceeded the annual totals for 2018 and 2019 (n = 232 and n = 311, respectively). In April 2020, the number of malaria deaths was more than triple the expected number based on previous years (Fig. 2B). The surge in malaria deaths was uneven and primarily observed in Mashonaland Central and Mashonaland West provinces. The number of malaria deaths reported between January and June 2020 exceeded the annual totals for 2018 and 2019 and the expected number by 28% (Table 1, Fig. 2B).

**Fig. 3:**
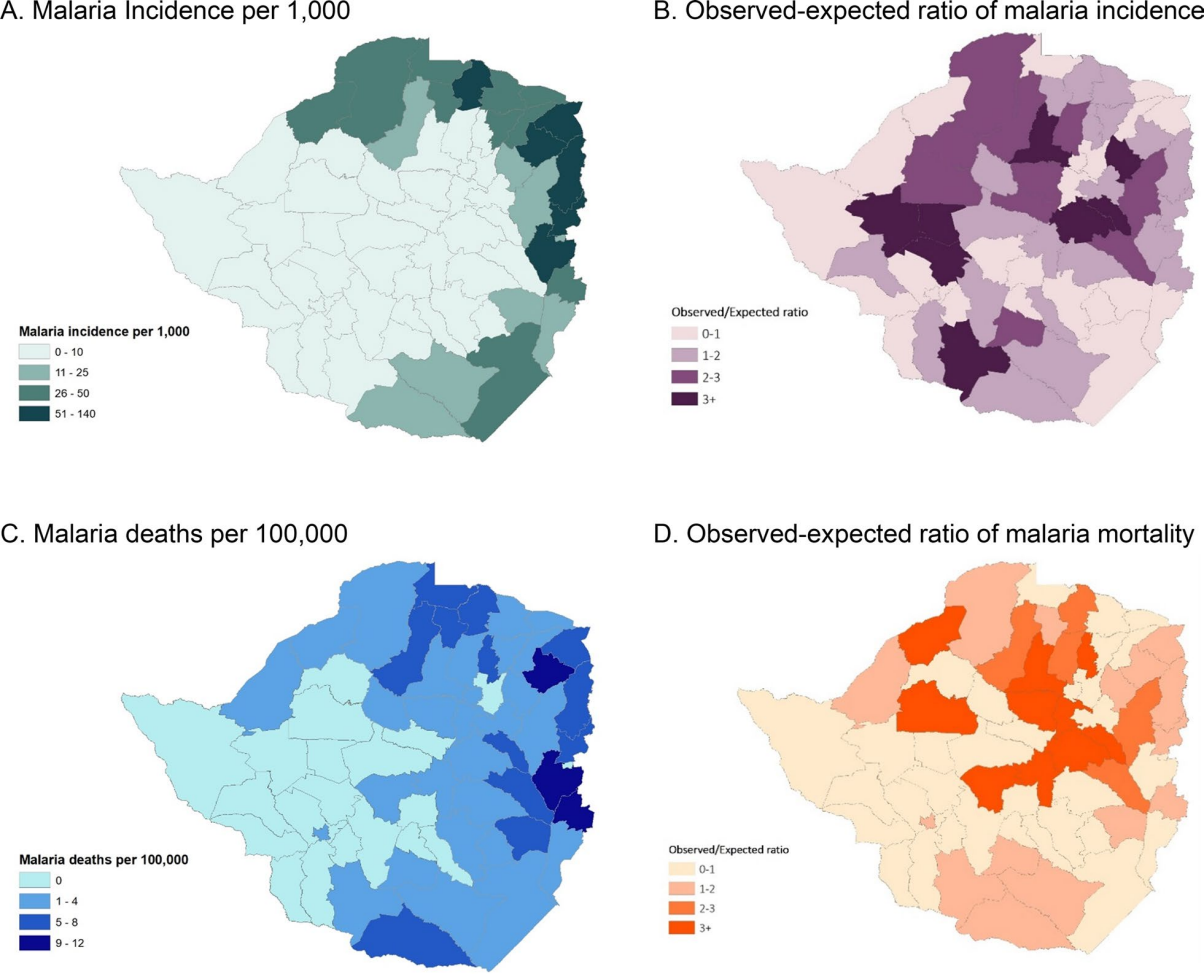
Map of malaria incidence and mortality in Zimbabwe, January–June 2020

Figure 3 shows the spatial variation of malaria incidence and mortality for January–June 2020. Districts along the northern, eastern and southern borders of the country reported the highest malaria incidence and mortality rates (Fig. 3). Out of 62 districts, 11 recorded higher than expected malaria incidence from January to June 2020, indicating possible malaria outbreaks (Fig. 3B). Strikingly, 8 districts (Bubi, Chikomba, Gwanda, Hwedza, Lupane, Murewa, Nkayi, and Zvimba) had malaria incidence rates more than triple the expected numbers for January to June 2020. Notably, only 3 of these districts were in the high malaria burden provinces of Manicaland, Mashonaland Central, Mashonaland East and Masvingo; the remaining 5 were in areas characterized by little to no malaria transmission. Similar patterns were observed for malaria mortality: 5 districts (Centenary, Gweru, Hwedza, Mazoe, Makonde) had more than triple the expected malaria mortality rate for January to June 2020 (Fig. 3C, D).

## Discussion

National efforts to control malaria have resulted in significant reductions in malaria morbidity and mortality in Zimbabwe. The reductions have been attributed to the provision of ITNs and IRS in high burden areas and improved diagnostics and effective anti-malarial treatment [29–31]. Despite reductions in recent years, there was a substantial increase in malaria cases and deaths in 2020. Consistent with the peak malaria transmission season in Zimbabwe, the number of malaria cases and deaths rose in January, peaked in April, then declined. From January-June 2020, there was a higher than expected increase in malaria cases and deaths compared to the 3 previous years (2017–2019). This increase resulted in an excess of over 30,000 malaria cases and 75 malaria deaths. Notably, the increase in malaria incidence and mortality rates was predominantly in districts outside of the high malaria burden provinces of Manicaland, Mashonaland Central, Mashonaland East and Masvingo, suggesting an increase in malaria transmission (Additional file 3: Figure S3).

There are several potential explanations for the apparent increase in malaria incidence and mortality rates observed between January and June 2020. The increase in malaria morbidity and mortality coincided with both the peak malaria season and the detection of the first cases of COVID-19 and implementation of nationwide containment and mitigation measures to curtail spread of COVID-19. It is likely that several factors explain the observed increase in malaria transmission and outbreaks, including COVID-19-related disruptions in the malaria prevention and control activities, limited availability of malaria commodities, modification of health-seeking behaviour, and possible changes in case management and reporting practices. According to WHO, less than half of the 22 million ITNs, that were expected to be distributed globally in 2020, had been distributed as of November 2020 [32]. Additionally, less than half of malaria-endemic countries that had planned IRS campaigns in 2020 had completed them [32]. Although it is likely the increased morbidity and mortality is the result of COVID-19-related disruptions in malaria prevention and control activities, for Zimbabwe it is unlikely to result from disruptions in IRS. IRS is typically deployed prior to the commencement of the rainy season in November/December. The NMCP began deploying for the 2019/2020 season in November 2019. A total of 2,151,375 rooms (88% of those targeted) were sprayed and a population of 3,164,344 (83% of targeted population) were covered [33].

Changes in health-seeking behaviour may have contributed to the observed surge in malaria cases and deaths. On one hand, lockdowns, curfews, in-country travel restrictions, and fear of infection may have disincentivized accessing routine health services resulting in the reduced uptake of essential health services and increased malaria transmission. In several malaria-endemic countries, including Zimbabwe, there was a decrease in malaria outpatient attendance in 2020 following the implementation of nationwide lockdowns [32]. On the other hand, the excess in malaria morbidity and mortality observed may be a result of efforts by the Ministry of Health and Child Care to maintain essential health services amid the pandemic. Individuals suspected of having malaria are routinely tested for malaria using rapid diagnostic tests (RDTs). Increased awareness and vigilance by health care workers may have improved and widened testing practices to diagnose malaria and reporting practices. Also, public health messaging encouraging early care-seeking for any COVID-19 symptoms, including fever, may have promoted prompt care-seeking. Given the overlapping symptoms between COVID-19 and malaria, individuals with fever, headache and body aches or weakness who under normal circumstances would not have sought care may have promptly sought care.

Nevertheless, it is likely that the surge in malaria cases and deaths is not entirely attributable to COVID-19 related disruptions. For example, the higher occurrence of malaria might be associated with variations in environmental factors such as temperature and rainfall. Previous research has showed the important role of environmental factors play in malaria transmission in Zimbabwe [34, 35]. To understand the drivers of the observed trends in malaria morbidity and mortality, further contextual information on the far-reaching impact of COVID-19 mitigation measures is needed. Furthermore, given the impact of the pandemic is likely to be lagged in time, further studies should consider time periods beyond the initial few months of the pandemic.

Several limitations are worth noting. First, malaria transmission is Zimbabwe is heterogenous, and the distribution of malaria risk even within districts is not uniform. Second, the analysis made use of routine malaria surveillance data, which are prone to data quality issues and low reporting rates. Although a data quality audit of the malaria data obtained from the national HMIS was not conducted in the present study, previous research suggests high data quality, timely reporting and high completeness [28]. Several research studies have used routine malaria surveillance data from Zimbabwe to assess changes in malaria indicators, such as intervention coverage and incidence rates [29, 30, 34]. Third, the observed trends in malaria incidence and mortality reported here likely do not represent trends in the 2020/2021 season. Lastly, this analysis only presented trends for two indicators: malaria incidence and malaria mortality. There is an opportunity to supplement data from the HMIS with data from household surveys assessing changes in health-seeking behaviour and intervention coverage and data from health facility surveys assessing availability and readiness of health facilities to provide essential malaria services [25].

## Conclusions

Limitations notwithstanding, this analysis described an excess of malaria morbidity and mortality which coincided with the onset of COVID-19 in Zimbabwe. While the drivers of the observed increases in malaria morbidity and mortality remain to be determined, the continued delivery of essential health services amid the COVID-19 pandemic remains crucial. Governments in low-middle income countries should prioritize continuity of essential health services to preserve the hard-earned gains realized through years of investments especially for communicable diseases like malaria.

## Supplementary Information

**Additional file 1: Figure S1.** Number of confirmed malaria cases by gender.

**Additional file 2: Figure S2.** Number of confirmed malaria cases by age.

**Additional file 3: Figure S3.** Number of malaria deaths by age.

## Data Availability

Data are available from the Zimbabwe National Malaria Control Programme. Requests for access to the data can be made to: Dr Joseph Mberikunashe (jmberikunashe@nmcpzim.co.zw).

## Abbreviations

ACT: Artemisinin-based combination therapy
COVID-19: Coronavirus disease 2019
DHS: Demographic and Health Surveys
HMIS: Health management information system
IRS: Indoor residual spraying
ITN: Insecticide-treated net
IPTp: Intermittent preventative treatment in pregnancy
MoHCC: Ministry of Health and Child Care
NMCP: National Malaria Control Programme
PHEIC: Public health emergency of international concern
RDT: Rapid diagnostic test
SMC: Seasonal malaria chemoprevention
WHO: World Health Organization
