# Supplementary figures and images for: Malaria incidence and mortality in Zimbabwe during the COVID-19 pandemic: analysis of routine surveillance data
Source: Malar J. 2021 May 24;20:233. doi: 10.1186/s12936-021-03770-7 (PMC8142064; doi:10.1186/s12936-021-03770-7)

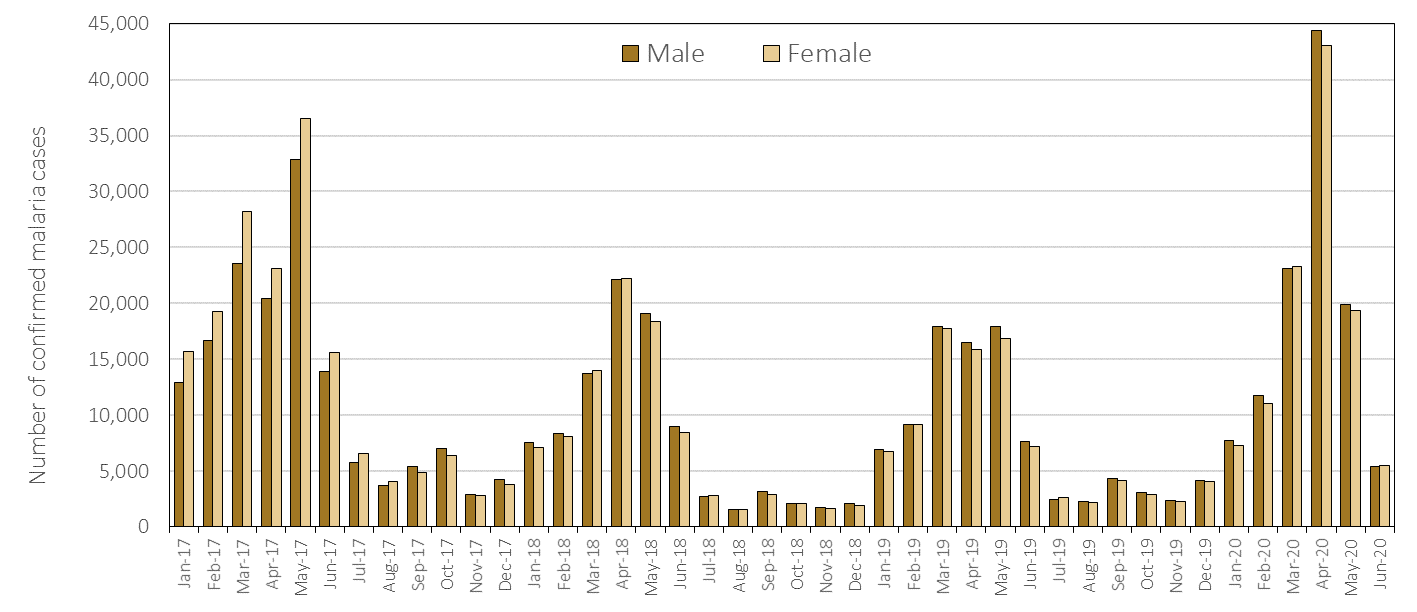

Supplement: Supplementary file 1 — Additional file 1: Figure S1. Number of confirmed malaria cases by gender. [file 12936_2021_3770_MOESM1_ESM.png]

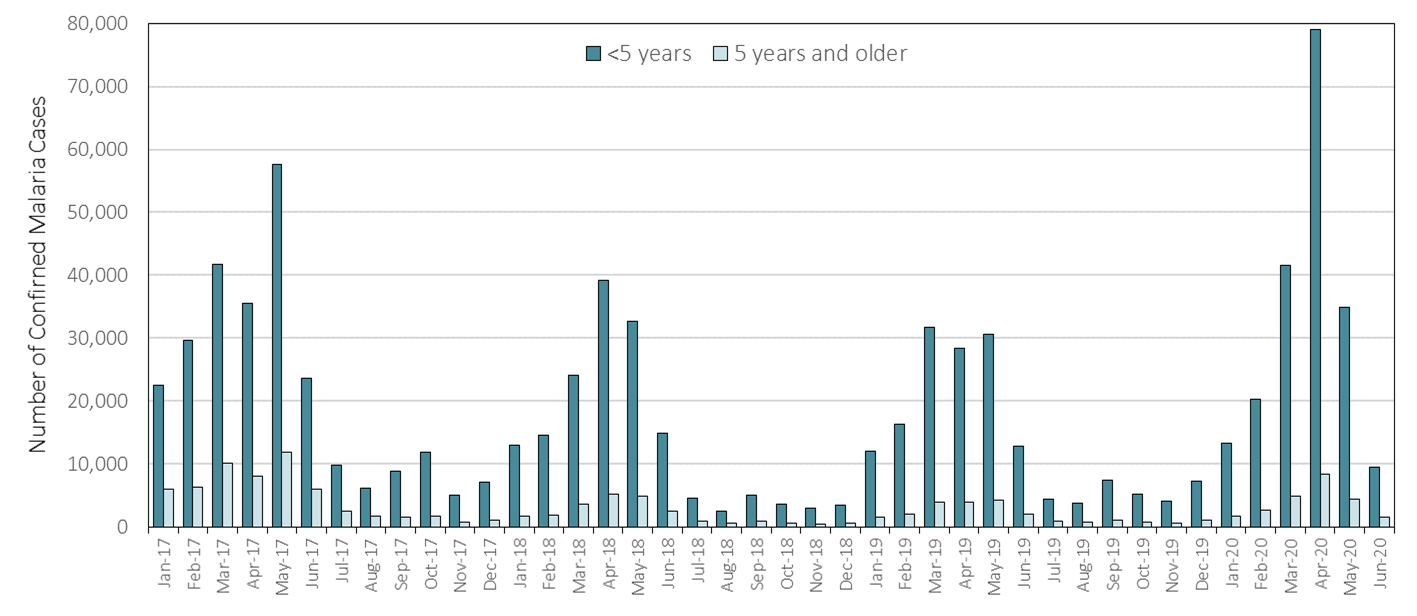

Supplement: Supplementary file 2 — Additional file 2: Figure S2. Number of confirmed malaria cases by age. [file 12936_2021_3770_MOESM2_ESM.png]

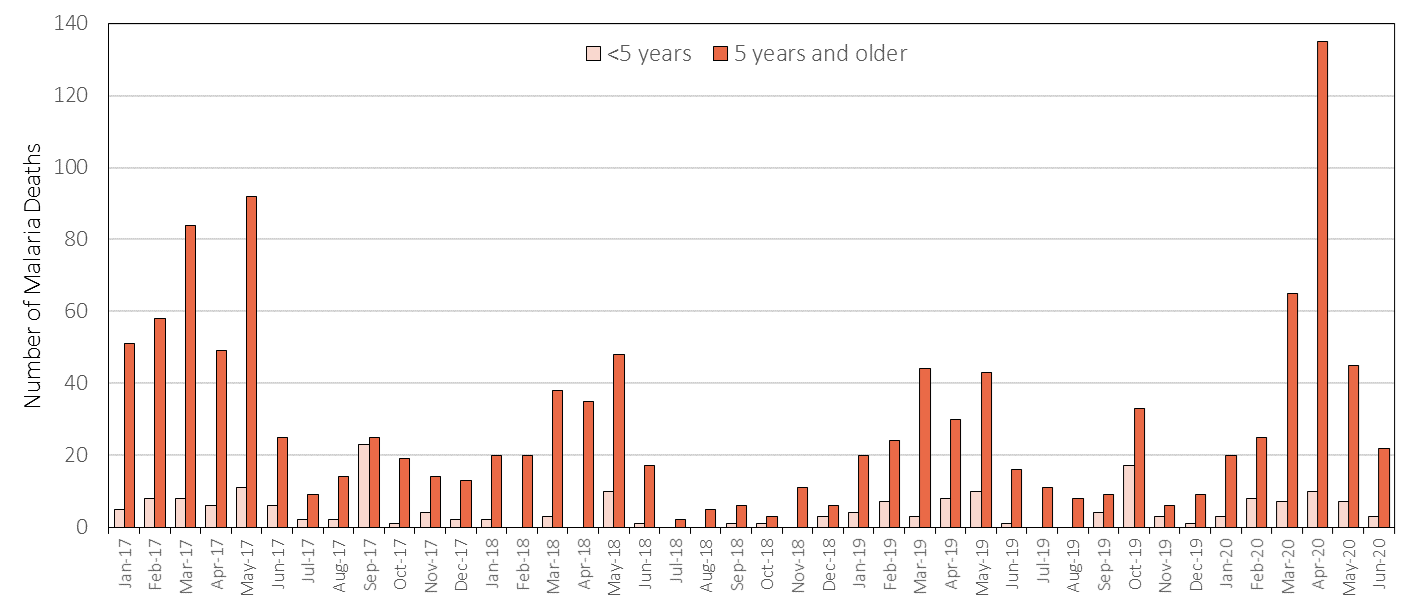

Supplement: Supplementary file 3 — Additional file 3: Figure S3. Number of malaria deaths by age. [file 12936_2021_3770_MOESM3_ESM.png]
